# The World’s Columbian Dental Congress

**Published:** 1892-06

**Authors:** 


### THE WORLD'S COLUMBIAN DENTAL CONGRESS.

We publish in the present number a full report of the decisions arrived at by the Executive Committee of this important meeting. Owing to an error in mailing, these failed to reach us in time for insertion in the May number.

The work of this committee has been of the most arduous character, involving unusual difficulties. That they have thus far given the preliminary arrangement most careful attention the report fully demonstrates. The list of names included in the committee's report embraces those of men prominent throughout the world, and involved very considerable labor in its preparation. There is much that is of importance yet to be done, and we anticipate from time to time to have the official announcements to lay before our readers.

While the committee are laboriously doing their share, it would be well for those who propose to prepare papers to be at work. These, it is needless to add, should be of a truly scientific character. At such a meeting nothing but original thought should be permitted.

